# Krüppel-like factor 4, a novel transcription factor regulates microglial activation and subsequent neuroinflammation

**DOI:** 10.1186/1742-2094-7-68

**Published:** 2010-10-15

**Authors:** Deepak K Kaushik, Malvika Gupta, Sulagna Das, Anirban Basu

**Affiliations:** 1 National Brain Research Centre, Manesar, Haryana-122050, India

## Abstract

**Background:**

Activation of microglia, the resident macrophages of the central nervous system (CNS), is the hallmark of neuroinflammation in neurodegenerative diseases and other pathological conditions associated with CNS infection. The activation of microglia is often associated with bystander neuronal death. Nuclear factor-κB (NF-κB) is one of the important transcription factors known to be associated with microglial activation which upregulates the expression of inducible nitric oxide synthase (iNOS), cyclooxygenase-2 (Cox-2) and other pro-inflammatory cytokines. Recent studies have focused on the role of Krüppel-like factor 4 (Klf4), one of the zinc-finger transcription factors, in mediating inflammation. However, these studies were limited to peripheral system and its role in CNS is not understood. Our studies focused on the possible role of Klf4 in mediating CNS inflammation.

**Methods:**

For *in vitro *studies, mouse microglial BV-2 cell lines were treated with 500 ng/ml *Salmonella enterica *lipopolysacchride (LPS). Brain tissues were isolated from BALB/c mice administered with 5 mg/kg body weight of LPS. Expressions of Klf4, Cox-2, iNOS and pNF-κB were evaluated using western blotting, quantitative real time PCR, and reverse transcriptase polymerase chain reactions (RT-PCRs). Klf4 knockdown was carried out using SiRNA specific for Klf4 mRNA and luciferase assays and electromobility shift assay (EMSA) were performed to study the interaction of Klf4 to iNOS promoter elements *in vitro*. Co-immunoprecipitation of Klf4 and pNF-κB was done in order to study a possible interaction between the two transcription factors.

**Results:**

LPS stimulation increased Klf4 expression in microglial cells in a time- and dose-dependent manner. Knockdown of Klf4 resulted in decreased levels of the pro-inflammatory cytokines TNF-α, MCP-1 and IL-6, along with a significant decrease in iNOS and Cox-2 expression. NO production also decreased as a result of Klf4 knockdown. We found that Klf4 can potentially interact with pNF-κB and is important for iNOS and Cox-2 promoter activity *in vitro.*

**Conclusions:**

These studies demonstrate the role of Klf4 in microglia in mediating neuroinflammation in response to the bacterial endotoxin LPS.

## Background

Inflammation in brain occurs in several neurodegenerative diseases and in response to pathogens invading the Central Nervous System (CNS). Several cell types have been implicated in inflammation mediated neurodegeneration, including microglia and astroglia [1]. Microglia, the resident macrophages of the CNS is the key cell type which responds to brain injuries or immunological stimuli such as endotoxins and becomes activated [2,3]. Upon activation, these cells release several pro- and anti-inflammatory cytokines in order to clear the pathogens and cellular debris from the brain tissue [4]. An exaggerated response by microglia, however, can result in neuronal damage indirectly by the secretion of cytokines or even directly by the phagocytosis of neurons [5] which can be detrimental to the normal functioning of the CNS [2]. Microglia is involved in inflammation initiated during several neurodegenerative diseases including Alzheimer's disease (AD) [6], Parkinson's disease (PD) and multiple sclerosis [7,8]. Understanding the molecular mechanisms of microglia-associated neuroinflammation has thus been central to many neurodegenerative and neuroinflammatory studies.

Microglial response to any pathogenic stimuli including endotoxins like lipopolysaccharide (LPS) is mediated by the activation of several pathways and numerous transcription factors. LPS is a major component of outer membrane of Gram-negative bacteria and it can elicit strong immune responses in the host [9]. LPS binds to toll-like receptor 4 (TLR4) on macrophages [10] and can activate nuclear factor-kappa B (NF-κB) [11-13] which is an important transcription factor in the regulation of pro-inflammatory enzymes and cytokines. LPS also mediates the production of inducible nitric oxide synthase (iNOS), cyclooxygenase-2 (Cox-2) and several pro- and anti-inflammatory cytokines via phospho NF-κB (pNF-κB) activation [14-16]. Recently, a class of zinc-finger transcription factors, Krüppel-like factors (Klfs) have been reported to play a key role in endothelial and macrophage mediated inflammation [17-19]. Klfs have three carboxyl C_2_H_2 _zinc-fingers in their DNA binding domain which has a strong homology with *Drosophila melanogaster *segmentation protein called Krüppel [20]. These three zinc fingers are usually found at the C-terminus and bind to either a CACCC element or GC-box [18]. The N terminus is involved in protein-protein interaction and gene regulation.

One of the Krüppel like transcription factors, gut Krüppel-like factor (GKLF) or Krüppel like factor-4 (Klf4) which was first identified in the epithelial lining of gut and skin is involved in differentiation and cellular growth [21,22]. Klf4 is also known to act as a key cell cycle regulator and a necessary mediator of p53 for the G1/S and G2/M cell cycle arrest resulting from DNA damage [23,24]. Klf4 also inhibits differentiation in murine stem cells [25], and induces pluripotency in mouse embryonic or adult fibroblasts [26]. In one of the studies, it was shown to transactivate iNOS promoter by potentially interacting with p65 of NF-κB in response to several inflammatory stimuli including LPS, tumor necrosis factor- α (TNF-α) and interferon- γ (IFN-γ) [18]. The expression of Klf4 significantly reduces upon treatment with anti-inflammatory tumor growth factor-β (TGF-β) and upon stimulation with neurotoxic agents [18,27]. Klf4 also binds to HMGB1 promoter, which is a cytokine mediator of systemic and local inflammations and is involved in facilitating its expression, translocation and release in RAW264.7 macrophages upon LPS stimulation [28]. However, the role of Klf4 in inflammation is ambiguous depending on the type of cell it is expressed in. For example, in endothelial cells, it is known to suppress inflammation [19] by binding to kallistatin, which in turn inhibits TNF-α mediated NF-κB activation and downregulates macrophage chemoattractant protein-1 (MCP-1) [29]. Lipopolysaccharide-induced endothelial nitric oxide synthase (eNOS), which is known to have anti-inflammatory activity, is significantly decreased upon Klf4 knockdown. In contrast to studies on human macrophages [18], in mouse monocytic cells, where Klf4 is shown to be involved in inflammatory monocyte differentiation *in vivo *and *in vitro*, Klf4 was not found to be involved in iNOS upregulation upon LPS stimulation [30]. All these studies indicate that Klf4 is pleiotropic in nature and its anti-inflammatory or pro-inflammatory functions are cell-type dependent. To date, however, there have been no studies in order to define the possible roles of Klf4 in microglial activation and neuroinflammation. Our studies, therefore, focus on its putative role in endotoxin-mediated microglial activation in brain.

In order to study the role of Klf4 in neuroinflammation, we treated mouse BV-2 cells with LPS to induce acute inflammation. Our data suggests that Klf4 expression increases with increasing dose or time of LPS treatment, and that this is accompanied by increased expression of iNOS, Cox-2 and several pro-inflammatory cytokines. Immunofluorescence and immunoblotting studies revealed that Klf4 translocates to nucleus after LPS treatment in both BV-2 as well as primary microglial cells. As suggested by knockdown studies, Klf4 plays a key role in augmenting the pro-inflammatory effects of LPS and causes microglial activation by upregulating iNOS and other pro-inflammatory cytokines including TNF-α, MCP-1 and IL-6. In addition to its role in mediating iNOS promoter activity, we also show for the first time that Klf4 is an important transcription factor required for Cox-2 promoter activity in response to LPS stimulation, thereby upregulating Cox-2 expression.

## Methods

### Cells and culture conditions

Mouse microglial cell line BV-2 was a kind gift from Dr. Steve Levison, University of Medicine and Dentistry, New Jersey, USA. The cell lines were grown at 37°C in Dulbecco's Modified Eagle's Medium (DMEM) supplemented with 5% sodium bi-carbonate (NaHCO_3_), 10% fetal bovine serum, penicillin at 100 units/ml and streptomycin at 100 μg/ml. All the reagents related to cell culture were obtained from Sigma Aldrich, St. Louis, USA, unless otherwise stated. Primary mixed glial cultures were prepared from P0-P2 mouse brains as described elsewhere [31,32]. Briefly, P0-P2 BALB/c mouse pups were sacrificed by decapitation and whole cortex was isolated. The meninges were removed and the tissues were enzymatically digested using trypsin-DNAse. After a brief mechanical dissociation, the cell suspension was passed through 100 mm cell strainers and then centrifuged at 400 g for 7.5 min. The cells were counted using hemocytometer and were plated into 75 cm^2 ^tissue culture flasks at a density of 2 × 10^5 ^viable cells/cm^2 ^in minimum essential medium (MEM) supplemented with 10% fetal bovine serum, penicillin at 100 units/ml, and streptomycin at 100 μg/ml, 0.6% glucose and 2 mM glutamine. Exhausted media was replenished with fresh media every 2-3 days after plating. On day 12, when the mixed glial culture was confluent, the flasks were shaken on an Excella E25 (New Brunswick Scientific, NJ, USA) orbital shaker at 250 rpm for 60-75 min to dislodge microglial cells. The non-adherent cells obtained after shaking were plated in bacteriological petridishes for 60-90 min to allow microglial cells to adhere. The adherent cells were scraped, centrifuged and plated in chamber slides at 8 × 10^4 ^viable cells/cm^2^, and incubated at 37°C for 30 min to allow microglial cells to adhere.

### LPS administration to animals

Three groups of 6-8 weeks old BALB/c mice were injected intraperitoneally with 5 mg/kg body weight of *Salmonella enterica *LPS dissolved in 1X PBS. Control animals received only 1X PBS. All the animals of each group were sacrificed at different time point of 3 h, 6 h, 12 h, 24 h and 48 h either for tissue or protein. All experiments were performed according to the protocol approved by the Institutional Animal Ethics Committee.

### Knockdown experiments

Short interfering RNA (SiRNA) against mouse Klf4 (sense: 5'- UCC AAA GAA GAA GGA UCU CUU- 3') and scrambled SiRNA (ScRNA) (sense: 5'- GUG CAC AUG AGU GAG AU UU- 3') were designed using an online SiRNA design software (Ambion, Applied biosystems, Austin, USA) and were synthesized by Dharmacon RNAi technologies (Thermo fisher Scientific, USA). 100 nM of Klf4 SiRNA was used for transfection using Lipofectamine RNAi max (Invitrogen, Carlsbad, CA, USA) according to the manufacturer's protocol. Briefly, BV-2 cells were seeded and maintained in sets of three at 37°C and 5% CO_2 _and when the cells were 70% to 80% confluent, they were transfected in Opti-MEM (Invitrogen) for 6 hours after which fresh 5% DMEM was added to the cells for 30 hours. The specificity of the antisense oligo was validated by using 100 nM of ScRNA and the control group (C) was treated with lipofectamine alone. The cells were then treated with 500 ng/ml of LPS for either 3 h for the analysis of changes in Klf4 mRNA levels by q(RT)-PCR or for 12 hours to analyse any changes in protein in order to perform immunoblotting and cytokine bead arrays (CBA).

### Luciferase assay

For luciferase assays, luciferase reporter gene constructs pGL2-iNOS (kindly provided by Dr. Mark A. Feinberg and Dr. Mukesh K. Jain, Harvard Medical School, Boston, MA, USA. The construct was originally made by Dr. Mark A. Perella, Harvard Medical School, Boston, MA, USA) [18,33] and pCOX301 (a kind gift from Dr. Manikuntala Kundu, Bose Institute, Kolkata, India) [34] were used that contained the 1,516 bp region from mouse iNOS 5'flanking region (-1485 bp to +31 bp region inserted in HindIII at 3'and KpnI at 5' sites which removes SV40 promoter upstream to luciferase gene from the construct) and Cox-2 promoter region (-891 bp to +9 bp region also inserted using HindIII at 3'and KpnI at 5' end) cloned upstream of luciferase reporter gene in pGL2-promoter and pGL2-basic vector respectively (Promega, Madison, WI, USA). After 24 hours of SiRNA and ScRNA transfection, 1 μg of these plasmids were used for transfection using 5 μl of lipofectamine 2000 (Invitrogen) in Opti-MEM for 6 hours. The cells were then maintained for additional 12 hours in 5% DMEM before the cells were treated with 500 ng/ml of LPS.

The luciferase assay was carried out using luciferase assay kit (Promega) according to manufacturer's protocol. Briefly, the cells were washed with 1X PBS and lysed in reporter lysis buffer provided by the kit and centrifuged at 12,000 g for 5 minutes at 4°C and supernatant was collected. 100 μl of luciferase assay reagent was dispensed into three sets of luminometer tubes and 20 μl of the collected supernatant was added to each tube and the reading was taken using Sirius single tube luminometer (Berthold detection systems GmBH, Germany). The luciferase units were measured as Relative Luciferase Units (RLU) and these values were normalized to the amount of protein present in the sample.

### Nitrite measurement

Nitrite generation by BV-2 cells was used as an indicator of NO release and measured by the Griess reaction as described elsewhere [31]. Briefly, the media from treated cells was centrifuged at 2,000 rpm for 5 min at 4°C to remove cellular debris. 50 μl of this media was incubated for 15 min at room temperature in dark with 50 μl of Griess reagent (sulfanilamide and *N*-1-napthylethylenediamine dihydrochloride) (Sigma Aldrich) under acidic (phosphoric acid) conditions in order to measure the nitrite content. The readings were taken spectrometrically at 540 nm using microplate spectrophotometer (Biorad, Australia) and the concentration was calculated from a sodium nitrite standard reference curve.

### Immunoblotting

For *in vivo *studies, the whole brain tissues (except olfactory lobes and hind brain) from three different animals after 3 h, 6 h, 12 h, 24 h and 48 h post LPS administration and untreated controls were dissected and placed in 1.5 ml microfuge tubes with 700 μl of freshly prepared 1X PBS containing 20 μl of protease inhibitor (Sigma Aldrich). Samples were homogenized and centrifuged at 8,000 g for 20 min. Supernatants were collected and protein concentrations were determined using a bicinchoninic assay (BCA) method.

For *in vitro *studies, control and treated BV-2 cells were harvested using ice-cold 1X PBS. In order to isolate total cellular extracts, the cells were lysed in buffer containing 1% Triton-X-100, 10 mM Tris-HCl (pH 8.0), 150 mM NaCl, 0.5% Nonidet P (NP-40), 1 mM EDTA, 0.2% EGTA, 0.2% sodium orthovanadate and protease inhibitor cocktail (Sigma Aldrich). For nuclear extracts, BV-2 cells were collected with 1X PBS and harvested by spinning at 2000 rpm for 5 min. The cells were resuspended in 400 μl of cold buffer A (10 mM HEPES pH 7.9, 10 mM KCl, 0.1 mM EDTA, 0.1 mM EGTA, 1 mM DTT and 0.5 mM PMSF) and then kept on ice for 15 min. 15 μl of nonionic surfactant, IGEPAL CA 630 (Sigma Aldrich) was then added and vortexed vigorously. The cells were then pelleted for 1 min at 10,000 g and the recovered pellet was resuspended in 50 μl of ice cold buffer B (20 mM HEPES pH 7.9, 400 mM KCl and 1 mM EDTA) and subjected to gentle shaking for 15 min at 4°C. The suspended cells in Buffer B were again pelleted at 4°C and the supernatant having the nuclear protein was collected and estimated by bicinchoninic assay (BCA) method.

30 μg of each protein sample was electrophoresed on a 7.5%-10% sodium dodecyl sulfate-polyacrylamide gel (SDS-PAGE) and transferred onto a nitrocellulose membrane. The membrane was then blocked in 5% skimmed milk in 1X PBS-Tween-20 (1X PBST) for 4 h at room temperature with gentle agitation. After blocking, the blots were incubated with rabbit anti-Klf4 (Chemicon International, CA, USA) at a dilution of 1:500 in 1% BSA in 1X PBST overnight at 4°C with gentle agitation. After five washes of five minutes each in 1X PBST, blots were then incubated with goat anti-rabbit horseradish peroxidase (Vector Laboratories, CA, USA) at a dilution of 1:5,000 in 1X PBST for 1.5 h, with agitation. The blots were rinsed again in 1X PBST. The blots were developed by using chemiluminescence reagent (Millipore, MA, USA) and exposing them to Chemigenius, Bioimaging System (Syngene, Cambridge, UK). The images were captured and analysed using the Genesnap and Genetools software respectively from Syngene. The blots were stripped for 30 min at 50°C in stripping buffer which contained 62.5 mmol/L Tris-HCl, pH 6.8, 2% SDS, 100 mmol/L 2-mercaptoethanol. The stripped blots were probed with anti-β-tubulin (1:1,000; Santa Cruz Biotechnology, CA, USA) or anti-β-actin (1:10,000; Sigma Aldrich) to determine equivalent loading of total cell extracts and nuclear extracts respectively. Similarly, blots were developed for rabbit anti-iNOS (1:1000, Millipore), Cox-2 (1:1000, Millipore) and pNF-κB (1: 1,000; Ser-536; Cell Signaling technology, MA, USA) using the secondary goat anti-rabbit HP (1:5,000, Vector Laboratories). The protein levels were normalized with either β-tubulin or β-actin levels.

### Immunocytochemistry

BV-2 cells and mouse primary microglial cells were plated on chamber slides in sets of three and allowed to adhere for 12 h. The cells were fixed in 4% PFA for 20 min at 25°C, following which they were incubated in blocking solution for 1 h at 25°C. They were then stained with rabbit anti-Klf4 (1:250, Millipore) overnight at 4°C. After PBS washes, the anti-rabbit FITC conjugated secondary antibody (1: 250, Vector Laboratories) were added for 1.5 h and then mounted with 4, 6-diamidino-2-phenylindole (DAPI). Images were obtained using Zeiss Apotome microscope (40X magnification; Zeiss, Germany).

### Immunohistochemistry

12 h and 48 h LPS treated and age matched control BALB/c mice in sets of three were perfused transcardially with 1X PBS and whole brains isolated were fixed with 4% paraformaldehyde (PFA) in 1X PBS for 24 h at 4°C and subsequently kept in 30% sucrose for additional 24-48 h at 4°C or until the tissue is immersed at the bottom of the tube. The brains were then processed for cryostat sectioning and the sections were stained for Klf4 and Iba1, a marker of activated microglia. Briefly, the cryostat sections were washed with 1X PBS and processed for antigen retrieval by incubating at 70°C for 1 h in antigen unmasking solution (Vector laboratories). The sections were then washed with 1X PBS and blocked for 1.5 h with 5% BSA in 1X PBS. Microglia were labeled with rabbit anti-Iba1 antibody (1:250; Wako, Japan), along with mouse anti-Klf4 (1:100, Santa Cruz biotechnology) by incubating the sections overnight at 4°C. After five washes with 1X PBS, the sections were incubated with goat anti-rabbit Alexa Fluor 594 (1:1000; Molecular Probes, Oregon, USA) for Iba1 and horse anti-mouse Fluorescein Isothiocyanate (FITC, 1:250; Vector Laboratories) for Klf4 for 1.5 h. The slides were then mounted with mounting medium containing DAPI (Vector laboratories). The stained slides were observed under the Zeiss Axioplan 2 Fluorescence microscope (20X magnification; Zeiss, Germany). Higher magnification images were captured with Zeiss LSM 510 confocal microscope (40X magnification; Zeiss, Germany).

### Co-immunoprecipitation

In order to study the interaction between pNF-κB and Klf4, co-immunoprecipitation was carried out using 100 μg of nuclear extract which was incubated with 1 μg of pNF-κB antibody (Cell signaling technologies) and 1 μg of rabbit IgG (Vector laboratories) in IP buffer containing 50 mM Tris-Cl pH 8.0, 150 mM NaCl, 10% glycerol, 1 mM Phenyl methyl sulfonyl fluoride (PMSF) and 0.5% Triton X-100 at 4°C overnight with gentle shaking. The Sepharose G beads (GE healthcare biosciences AB, Uppsala, Sweden) were calibrated with IP buffer and 30 μl of the slurry was then added to the protein-antibody mixtures for 4 h at room temperature with gentle rocking. The beads were then pelleted at 12,000 g for 2 min and washed four times with 600 μl of ice cold IP buffer after discarding the supernatant. The sample buffer was then added to each sample bead and loaded onto gel for immunoblotting.

### Reverse transcriptase PCR (RT-PCR) and quantitative real-time (qRT)-PCR

BV-2 cells, following treatment were washed twice with 1X PBS and lysed in Trizol reagent (Sigma Aldrich) as per the manufacturer's protocol. The RNA was isolated by phenol-chloroform method and it was quantified using spectrophotometer (GE healthcare biosciences AB, Uppsala, Sweden). RT-PCR was performed using the onestep RT-PCR kit (Qiagen Biosciences; Hamburg, Germany) following the manufacturer's protocol on Genius Techne thermal cycler as described earlier [35]. Briefly, 500 ng of the total RNA was used as template in 25 μl PCR reactions, containing 5X PCR buffer (5 μl), dNTPs (1 μl), enzyme mix (1 μl) and specific forward and reverse primers (0.5 μM) and RNase free water. Oligonucleotide primer specific for mouse iNOS (forward: 5'-CCC CCG AAG TTT CTG GCA GCA GC-3', reverse: 5'-GGC TGT CAG AGC CTC GTG GCT TTG G -3', annealing temperature: 62°C, 35 cycles, amplicon size: 468 bp), Cox-2 (forward: 5'-AAG GCC TCC ATT GAC CAG -3', reverse: 5'- TCT TAC AGC TCA GTT GAA CGC -3', annealing temperature: 56°C , 35 cycles, amplicon size: 512 bp) and cyclophilin mRNAs (forward: 5'-CCA TCG TGT CAT CAA GGA CTT CAT -3', reverse: 5'-TTG CCA TCC AGC CAG GAG GTC T -3', annealing temperature: 58°C , 35 cycles, amplicon size: 192 bp) were procured from Microsynth (Balgach, Switzerland). PCR products were separated on 1.5% - 2% agarose gel, stained with ethidium bromide, and photographed using GeneSnap software provided with Chemigenius Bioimaging System, Syngene. Photographs were analyzed by GeneTools software provided with same Bioimaging System. The mRNA levels were normalized to cyclophilin.

For performing q(RT)-PCR for Klf4 mRNA, Klf4 primers (forward: 5'-TGC CAG ACC AGA TGC AGT CAC- 3', reverse: 5'-GTA GTG CCT GGT CAG TTC ATC- 3', annealing temperature: 60°C, 35 cycles, amplicon size: 286 bp) were procured from Sigma. SYBR Green Supermix (Bio-Rad, Hercules, CA) was used and 500 ng of cDNA was used as a template on ABI Prism 7700 sequence detection system (Applied Biosystems, Foster City, CA). The conditions used for real time PCR have been described earlier [36] and were as follows: 95°C for 3 min (1 cycle), 94°C for 20 s, 60°C for 30 s, and 72°C for 40 s (40 cycles). The dissociation curves were generated to check for the specificity of primer annealing to the template. The real time PCR results were normalized to 18S rRNA internal control and quantified using comparative C_t _method (2^-[∆][∆]Ct^) [37] and analyzed using the iCycler Thermal Cycler Software (Applied Biosystems).

### Cytokine bead array (CBA)

A CBA mouse inflammation kit (BD Biosciences, NJ, USA) was used to quantitatively measure cytokine levels in the control and LPS treated BV-2 cells. Using 50 μl of mouse inflammation standard and sample dilutions, the assay was performed according to the manufacturer's instructions and analyzed on the FACS Calibur (Becton Dickinson). This method quantifies soluble particles, in this case cytokines using a fluorescence based detection mechanism. The beads, coated with IL-6, TNF-α and MCP-1 react with test lysates and standards, to which fluorescence dyes are then added. Analysis was performed using CBA software that allows the calculation of cytokine concentrations in unknown lysates [35,38].

### Electrophoretic mobility shift assay (EMSA)

EMSA was performed as described elsewhere [39] using LightShift Chemiluminescent EMSA Kit (Pierce Biotechnology, Thermo Fisher Scientific Inc., Rockford, USA). Briefly, 100 femtomoles of biotin labeled and 100-molar excess of unlabelled Oligonucleotide (Cold probe) corresponding to iNOS-binding sequence (5- CTG CCT AGG GGC CAC TG -3) were used (Sigma Proligo, Singapore). Nuclear extract (5 μg) isolated from LPS-treated BV-2 cells was pre- incubated with anti-Klf4 Gel shift transcruze antibody (1 μg; Santa Cruz Biotechnology) for 1 h at 4°C. Biotin-labeled probes were added for additional 30 min at room temperature. After electrophoresis on 6% polyacrylamide gel, the samples on gel were transferred onto a presoaked positively charged nylon membrane (Roche diagnostics, GmBH, Germany). The ultra violet cross linking was carried out using UV cross linker (UVC 500, Hoefer scientific, USA). The blots were then incubated in blocking buffer for 15 min followed by incubation with streptavidin-horseradish peroxidase (1:300; HRP) conjugate solution for additional 15 minutes with gentle shaking. After washing with wash buffer supplied by the manufacturer, the membrane was then incubated in substrate equilibration buffer for five min followed by addition of Enhanced Chemiluminiscent reagent (ECL) supplied by the manufacturer. The blot was developed by exposing it to Chemigenius, Bioimaging System (Syngene).

### Statistical Analysis

All the experiments were performed in sets of three unless otherwise mentioned and the data generated were analyzed statistically by paired two-tailed Student's t-test. A statistical p-value of 0.01 and 0.05 were considered significant.

## Results

### Dose and time dependent increase in Klf4 levels in BV-2 cells in total cell extract

**Figure 1:**
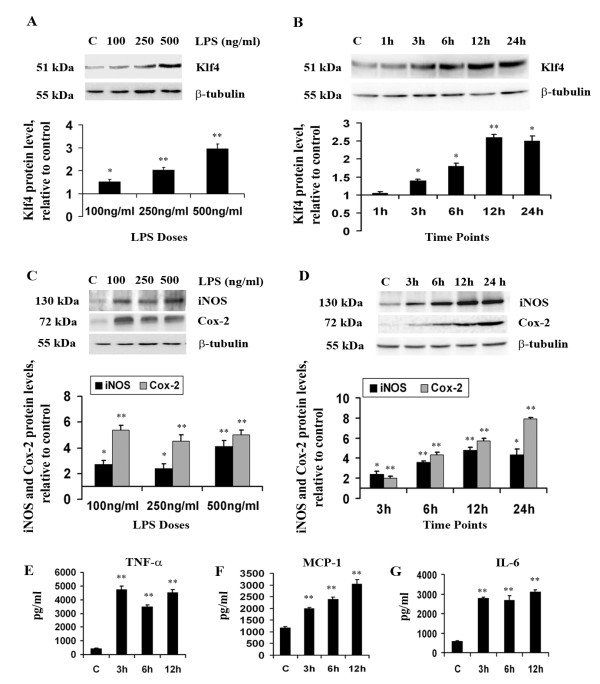
**Klf4 expression and inflammation in BV-2 microglial cells upon LPS stimulation**. Total cellular extract isolated from cells treated with different doses of LPS and for different time points were analyzed by immunoblot. **(A and B) **A significant increase is seen in Klf4 expression in a dose-dependent **(A) **and time dependent-manner **(B) **compared to untreated control samples. (**C and D) **There are significant increases in iNOS and Cox-2 levels in dose- **(C) **and time-dependent **(D) **manners in cells treated with LPS. The graphs represent protein levels relative to untreated controls. **(E-G) **Increase in pro-inflammatory cytokines upon LPS stimulation. Cytokine bead arrays were carried out to estimate the concentrations of TNF-α **(E)**, MCP-1 **(F) **and IL-6 **(G) **in BV-2 cells treated for different time points. There were significant increases in all of these pro-inflammatory cytokines upon LPS treatment. Absolute values of these cytokines are given as pg/ml. *, **, Statistical differences in comparison to control values (* p < 0.05; ** p < 0.01).

Any changes in Klf4 in microglia in response to endotoxins have not been reported till now. Therefore, we investigated whether mouse microglial BV-2 cells express Klf4 and how its expression profile changes in response to LPS treatment. BV-2 cells were treated with LPS in a dose- and time-dependent manner in order to detect any change in the expression levels of Klf4. For dose-dependent studies, three different doses of 100 ng/ml, 250 ng/ml and 500 ng/ml of LPS were given for 12 hours. Increased expression of Klf4 was observed at 100 ng/ml dose, and it gradually increased with higher doses, the maximum expression being at 500 ng/ml LPS where a 3-fold increase was observed over control, (p < 0.01) (Fig 1A). Since this dose is in agreement with many inflammation studies, we therefore chose 500 ng/ml LPS for carrying out treatments in the rest of our experiments. Five different time points of 1 h, 3 h, 6 h, 12 h and 24 h were chosen for time dependent studies for the analysis of Klf4 expression. We observed that there was no significant change in the levels of Klf4 expression in total cellular extracts after 1 h of LPS treatment, however a gradual increase was observed at later time points. After 3 hours of LPS treatment an increase of about 1.5 times to that of control was seen (p < 0.05) and the levels of Klf4 expression were found to be maximum at 12 h and 24 h time points when more than 2.5-fold increase over that of control was observed (p < 0.01) (Fig 1B).

### LPS treatment increases the expression of inflammatory mediators, iNOS, Cox-2 and pro-inflammatory cytokines in BV-2 mouse microglial cells

In order to confirm the onset of inflammation in BV-2, we next investigated the expression levels of pro-inflammatory effector enzymes, iNOS and Cox-2 in a dose and time-dependent manner upon LPS treatment. This study was carried out using the same doses and four different time points except for 1 h time point where we did not notice any considerable change in Klf4 expression. We observed a gradual increase in the levels of both iNOS and Cox-2 with increasing dose and time of LPS treatment. Significant 4-fold increase in iNOS expression with 500 ng/ml LPS dose after 12 hours of stimulation was observed which was found significant with respect to control (p < 0.01) (Fig 1C). The expression of iNOS increased at earlier time points of 3 and 6 hours of LPS treatment and this increase was also found to be significant with respect to control (p < 0.01) (Fig 1C). Similarly, Cox-2 expression also increased by more than 5-fold with respect to control after 12 hours of LPS treatment (p < 0.01) (Fig 1D). This increase in iNOS and Cox-2 levels were found to be significant even after 24 hours of LPS stimulation (p < 0.01). Microglial activation and neuroinflammation is also associated with the release of several pro-inflammatory cytokines and chemokines. CBA using BV-2 cell lysates was performed to test any changes in the levels of key pro-inflammatory cytokines and chemokines, TNF-α, MCP-1 and IL-6 upon LPS stimulation. We observed a significant increase the levels of these mediators with increasing time point. The levels of TNF-α, an acute phase protein that is secreted by macrophages and microglia in response to pathogenic stimuli, increased by more than 10-folds and reached up to 5,000 pg/ml (p < 0.01) at 3 h which was significant with respect to control and by 12 h, the levels were maintained at 4,000 pg/ml (p < 0.01) (Fig 1E). MCP-1, a chemokine which is known to recruit inflammatory cells into CNS parenchyma [40], was recorded to be around 3,000 pg/ml at 12 h of LPS stimulation which was 3 times more than control levels (p < 0.01) (Fig 1F). Another pro-inflammatory cytokine, IL-6 which is pleotropic in nature [41,42] is known to increase dramatically in response to inflammation and CNS injury. The levels of IL-6 also increased to 4,000 pg/ml in response to LPS stimulation which is an increase of about 4-fold with respect to control at all the time points (p < 0.01) (Fig 1G). We observed that the increase in expression levels of Cox-2, iNOS and other pro-inflammatory cytokines were corresponding to the levels of Klf4 which indicates that this transcription factor might have a role in mediating inflammatory response in BV-2 following LPS stimulation.

### Increase in nuclear Klf4 upon LPS stimulation *in vitro*

**Figure 2:**
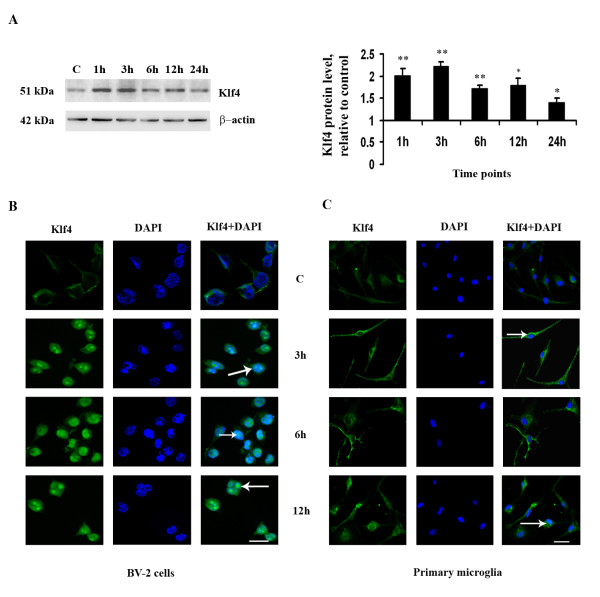
**Nuclear expression and translocation of Klf4 in response to LPS**. Immunoblotting was carried out for nuclear extracts from LPS-treated BV-2 cells in a time-dependent manner. **(A) **A significant increase is observed in Klf4 levels in nuclear extracts at the 1 h and 3 h time points. The graph represents nuclear Klf4 protein levels relative to untreated controls. *, **, Statistical differences in comparison to control values (* p < 0.05; ** p < 0.01). For nuclear translocation studies, immunofluorescence for Klf4 was carried out in BV-2 cells and primary microglia. **(B) **Increased expression and nuclear translocation of Klf4 in BV-2 cells at 3 h, 6 h and 12 h time points, indicated by arrows. **(C) **Increased expression of Klf4 in mouse primary microglia cells and nuclear localization of Klf4 at the 12 h time point are indicated by arrows. Scale bar: 20 μm.

In order to evaluate Klf4 levels in nucleus following LPS stimulation, nuclear extract from BV-2 cells treated with LPS for different time points of 1 h, 3 h, 6 h, 12 h and 24 h was isolated and analyzed for Klf4 protein expression by performing western blots. A significant 2-fold increase in Klf4 levels was observed at 1 h and 3 h time points compared to control (p < 0.01) (Fig 2A). The increased level of Klf4 was maintained even at later time points with more than 1.5-fold increase even at 12 h time point (p < 0.05).

In order to study the nuclear translocation of Klf4, we carried out immunostaining in LPS treated BV-2 microglial cell line and mouse primary microglia for different time durations. We found that in BV-2 cells Klf4 translocates to nucleus as early as 3 h which can be seen till 12 h (Fig 2B). It is seen localized to specialized regions in the nucleus which probably are transcriptionally active regions or nucleoli. Similarly, in primary microglia, after 3 h and 6 h of LPS treatment, the expression of Klf4 increases and is perinuclear in location which at 12 h time point translocates to nucleus (Fig 2C). This study reveals that Klf4 migrates to nucleus at earlier time points and as a transcription factor probably has a role in onset of inflammation.

### LPS induces Klf4 expression *in vivo*

**Figure 3:**
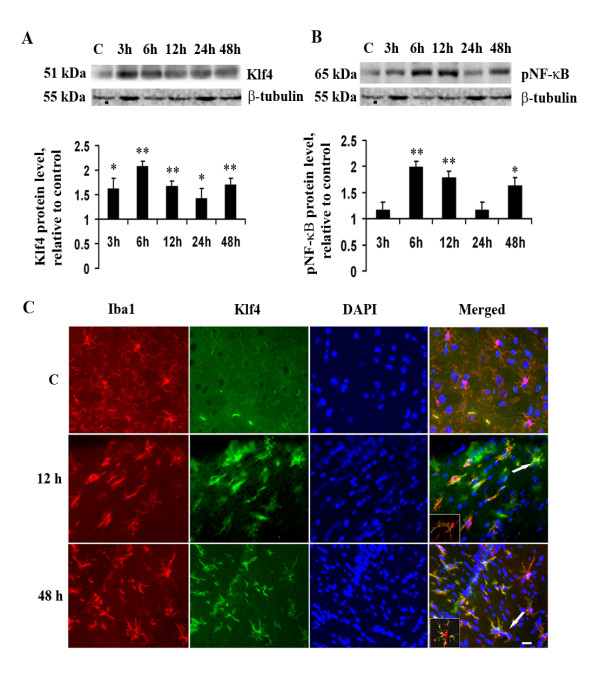
**Klf4 expression *in vivo *upon LPS administration**. Immunoblot and immunohistochemistry analysis of Klf4 expression in BALB/c mice brains upon LPS administration. **(A) **There are significant increases in Klf4 expression at different time points compared to control brain. **(B) **There is a significant increase in pNF-κB levels upon LPS administration in a time-dependent manner compared to control brain. The graphs represent pNF-κB and Klf4 protein levels in brains of LPS-treated mice relative to control mouse brain. *, **, Statistical differences in comparison to control values (* p < 0.05; ** p < 0.01). **(C) **Fluorescent microscopy images of Klf4 expression in microglial cells in brain cortices of control and LPS-treated mice. Klf4 (FITC, green) co-localizes with Iba1- (Alexa Fluor 594, red) positive microglial cells at 12 h and 48 h after LPS administration. Co-localization is shown by arrows in the merged images. **Inset**. The *inset *depicts high resolution confocal images of Klf4 and Iba1 co-localization (yellow) in single cells at the 12 h and 48 h time point. Data represent groups of 3 animals per treatment. Scale bar: 50 μm.

We wanted to investigate if LPS treatment increases Klf4 expression in microglia *in vivo *as well. Increase in Klf4 expression was observed in total protein extracts from the brains of mice treated with LPS over control. This increase, although, was not gradual with increasing time points but was significantly higher at all the time points with respect to control (Fig 3A). At 6 h time point, 2-fold increase in Klf4 expression with respect to control was observed (p < 0.01) (Fig 3A). This increase corresponded with the elevated levels of pro-inflammatory cytokines from these mice (data not shown). We also estimated the levels of pNF-κB at all the time points and found that its levels significantly increased over that of control with maximum increase noticed at 6 h and 12 h (p < 0.01) (Fig 3B). Immunohistochemistry also confirmed the expression of Klf4 in microglial cells. Numerous Iba1 positive cells with activated morphology were observed in LPS treated sections at all time points. Analysis of 12 h and 48 h brain sections from mice treated with LPS revealed the co-localization of Klf4 with Iba1 positive microglia (Fig 3C). Individual cells imaged using high resolution confocal microscopy also confirms Klf4 expression in these Iba1 positive cells (Inset).

### Knockdown of Klf4 and subsequent decrease in production of pro-inflammatory cytokines

**Figure 4:**
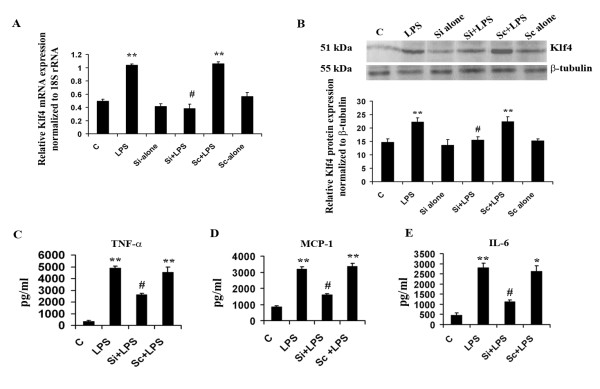
**Role of Klf4 in mediating inflammation**. Knockdown of Klf4 in BV-2 mouse microglial cells using SiRNA against Klf4 mRNA and subsequent decrease in the expression of pro-inflammatory cytokines. **(A) **q(RT)-PCR demonstrate a significant decrease in Klf4 mRNA levels in Si+LPS cells compared to LPS alone-treated cells. Cells treated with lipofectamine alone served as controls. The graph represents relative Klf4 mRNA expression values normalized to 18S rRNA internal control. **(B) **Immunoblot analysis of Klf4 protein isolated from BV-2 cells. Klf4 protein levels were decreased significantly in the Si+LPS group compared to LPS alone and to the Sc+LPS group. The graph represents Klf4 protein levels normalized to β-tubulin. No significant differences were observed in Si-alone and Sc-alone conditions compared to control cells for both mRNA and protein levels. **(C-E) **Cytokine bead array analysis of pro-inflammatory cytokines upon Klf4 knockdown. There is a more-than-two-fold decrease in TNF-α **(C) **and MCP-1 **(D) **levels in Klf4 knockdown samples, and a significant three-fold decrease is noticed in the case of IL-6 **(E)**. *, **, Statistical differences in comparison to control values (* p < 0.05; ** p < 0.01) and #, Statistical differences with respect to LPS treated values, (# p < 0.01).

In order to confirm the role of Klf4 in mediating inflammation, we carried out knockdown of Klf4 transcription factor using SiRNA directed against its mRNA in BV-2 cells in both LPS (Si+LPS) treated and untreated (Si-alone) conditions. Cells were also transfected with ScRNA in both LPS treated (Sc+LPS) and untreated (Sc-alone) sets. To confirm the knockdown, we performed a quantitataive Real Time (qRT)-PCR for Klf4 mRNA along with an immunoblot for Klf4 and found a significant more than 2.5-fold decrease in Klf4 mRNA in SiRNA transfected samples upon LPS treatment (Si+LPS) compared to LPS alone treated cells (p < 0.01) (Fig 4A). Significant 1.4-fold decrease was also confirmed at the protein level where the Klf4 protein level was significantly reduced in Si+LPS cells (p < 0.05) (Fig 4B) with respect to LPS treated cells. No significant changes in Klf4 mRNA or protein level was noticed upon LPS treatment in ScRNA transfected (Sc+LPS) with respect to LPS alone condition. There was no significant change observed in either Si-alone or Sc-alone treatments with respect to untreated control cells and their values were found to be comparable to these controls.

Considering the potential role of Klf4 in inflammation, we hypothesized that Klf4 may play a role in the upregulation of cytokines such as TNF-α, MCP-1 and IL-6. Since there was no significant change in Klf4 levels in either Si-alone or Sc-alone conditions with respect to untreated controls, rest of the experiments did not include these conditions and only untreated cells served as control. CBA analysis was carried out to evaluate the levels of these cytokines after 12 hours of LPS treatment in BV-2 cells. As we have noticed earlier, the levels of all the cytokines in LPS treated cells were significantly elevated with respect to that of control. However, in Si+LPS cells, a significant reduction in their levels with respect to LPS and Sc+LPS cells was found. TNF-α levels were significantly reduced by about 2-fold in Si+LPS cells (p < 0.01) (Fig 4C). A similar 2-fold reduction was also observed in MCP-1 levels in Si+LPS cells transfected samples when compared to LPS alone treated cells (p < 0.01) (Fig 4D). The CBA also revealed a significant reduction by more than 2-fold in IL-6 levels when compared to Sc+LPS and LPS alone treated samples (p < 0.01) (Fig 4E). We can therefore conclude from these observations that Klf4 plays an important role in mediating inflammation as knocking it down significantly reduces the production of pro-inflammatory cytokines in BV-2 microglial cells.

### Klf4 knockdown results in decreased iNOS expression and NO production

**Figure 5:**
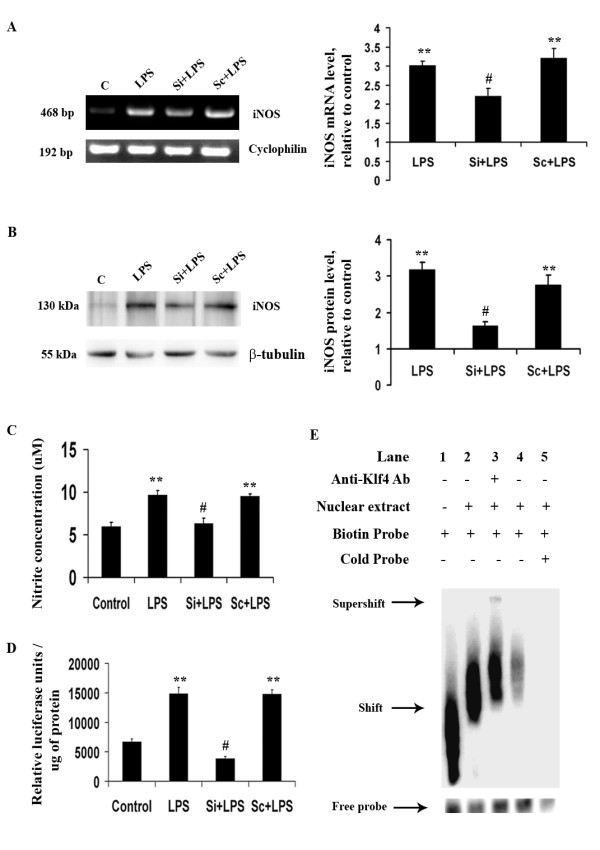
**Role of Klf4 in iNOS expression upon LPS stimulation**. **(A) **RT-PCR for iNOS mRNA demonstrates a significant decrease in iNOS mRNA levels upon Klf4 knockdown compared to LPS-treated cells. **(B) **Immunoblot showing iNOS levels under different conditions. There is a significant decrease in iNOS proteins levels in Si+LPS cells compared to LPS-treated cells. The graphs represent relative iNOS mRNA and protein levels with respect to the untreated controls. **(C) **Nitrite assay using Griess reagent was carried out to measure iNOS activity. A significant reduction is noticed in NO production as a result of Klf4 knockdown in LPS-treated samples. **(D) **Luciferase assay for iNOS promoter activity. Data is represented as relative luciferase units/amount of protein (in μg). A more-than-3-fold decrease is observed in luciferase activity in Si+LPS cells compared to LPS-treated cells. **(E) **EMSA carried out with nuclear extracts of control and LPS-treated BV-2 cells. Lane 1 shows free iNOS probe, whereas a shift is noticed in lane 2 when nuclear extracts were incubated with the probe. Lane 3 shows a supershift when Klf4-specific antibody was incubated with the probe and the nuclear extracts. The shift and supershift are indicated by arrows. Lane 4 shows a decreased shift when nuclear extracts from unstimulated control BV-2 cells were incubated with the iNOS probe. In lane 5, in addition to biotinylated probe, a 100-molar excess of unbiotinylated (Cold) probe was added along with nuclear extracts from LPS-stimulated cells. No significant band is observed in this lane. *, **, Statistical differences in comparison to control values and #, Statistical differences with respect to LPS-treated values respectively (* p < 0.05, ** p < 0.01, # p < 0.01).

Klf4 regulates iNOS levels in human macrophages [18]. However, its role in microglial activation is not known, we therefore, wanted to evaluate iNOS expression upon Klf4 knockdown in BV-2 cells. We carried out semi-quantitative RT-PCR in order to estimate the iNOS mRNA levels. Since we did not observe any significant change in the levels of Klf4 in Si-alone and Sc-alone conditions, we transfected cells with both SiRNA and ScRNA and treated them with LPS. A significant 2-fold decrease in iNOS mRNA levels in Si+LPS cells was found with respect to LPS and Sc+LPS cells (p < 0.01) (Fig 5A). We also noticed a significant decrease in iNOS protein levels as confirmed by immunoblot analysis which showed significant 2-fold over LPS treated samples (p < 0.01) (Fig 5B). Since Klf4 knockdown resulted in decreased production of iNOS, we also estimated the levels of nitric oxide (NO) a product generated by iNOS. NO was measured by estimation of nitrites (a stable product of NO) in the supernatants from differently treated BV-2 cells. The colorimetric analysis of nitrite production was carried out by adding Griess reagent to these supernatants. The concentration of nitrite in cell supernatants of LPS and Sc+LPS cells was markedly elevated after 12 h of LPS treatment (p < 0.05) (Fig 5C). We noticed that Klf4 knockdown resulted in a 2-fold reduction in NO levels (p < 0.05). This indicates that Klf4 regulates the expression of iNOS and its inflammatory mediators.

### Klf4 regulates iNOS promoter activity in BV-2 microglial cells

Klf4 has been shown to bind to the iNOS promoter in human macrophages upon LPS and IFN-γ treatment [18]. Since we saw a decrease in iNOS levels upon Klf4 knockdown in microglial cells and that iNOS is mostly regulated at the transcriptional level [43], we wanted to know if Klf4 also interacts with iNOS promoters in microglial cells upon endotoxin stimulation. Interaction of Klf4 to iNOS promoters was assessed using iNOS-pGL2 luciferase construct that has firefly luciferase directly driven by iNOS promoter. This assay was carried out for untreated control as well as LPS, Si+LPS and Sc+LPS treated BV-2 cells. We found that in LPS and Sc+LPS treated cells, the promoter activity measured in terms of relative luciferase units increased significantly with respect to control (p < 0.01) but decreased significantly by 5-folds in Si+LPS treated cells (p < 0.01) (Fig 5D). This finding suggests that Klf4 is an important transcription factor required for iNOS promoter activity and considering its role as a transcription factor, it may interact with iNOS promoter in microglial cells upon LPS stimulation.

In order to confirm this, we then carried out electromobility shift assay (EMSA) using the biotinylated iNOS probe for -212 bp position of iNOS promoter (5- CTG CCT AGG GGC CAC TG -3). This sequence was found to be important for Klf4 binding to iNOS promoter and it is close to pNF-kB binding site on this promoter [18]. In this experiment, nuclear extracts were isolated from 12 h LPS treated sample and incubated with the biotinylated iNOS probe along with or without the cold probe. This resulted in a 'shift' (Lane 2) with respect to probe alone (Lane 1). Addition of anti-Klf4 antibody resulted in 'supershift' with respect to the shift observed in protein-DNA complex (Lane 3; Fig 5E). We also carried out EMSA with nuclear extracts from unstimulated control cells and observed a significant decrease in the 'shift' which confirms that the iNOS probe binding increases upon LPS stimulation (Lane 4). In order to confirm the specificity of Klf4 for the probe sequence, we also incubated nuclear extracts in presence of 100-molar excess of cold probe and observed a significant decrease in the intensity of shifted bands (Lane 5) which indicate that this probe is rather specific in nature. We can hereby conclude from these experiments that Klf4 interacts with iNOS promoter upon LPS induction. This is how Klf4 may be directly upregulating iNOS production at the transcriptional level itself thereby influencing the production of NO.

### Klf4 also regulates Cox-2 expression in microglia in response to LPS

**Figure 6:**
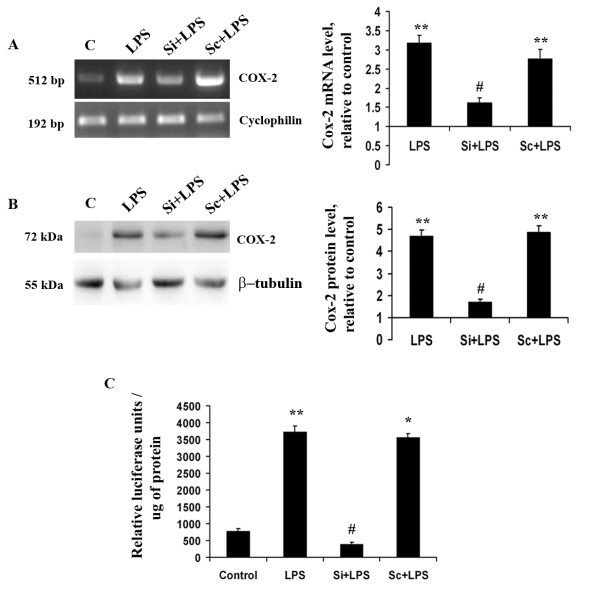
**Role of Klf4 in Cox-2 expression**. **(A) **Semi-quantitative RT-PCR of Cox-2 mRNA indicates a significant decrease in Si+LPS cells compared to LPS-treated cells **(B) **Immunoblot for Cox-2 in total protein isolates from BV-2 cells. The protein levels of Cox-2 were also found to be significantly reduced within 12 h of LPS treatment in Si+LPS cells compared to LPS-treated cells. The graphs represent relative Cox-2 mRNA and protein levels with respect to the control samples. **(C) **Luciferase assay for Cox-2 promoter activity using a pCOX301/pGL2 construct. This construct has the luciferase gene directly regulated by Cox-2 promoter. There is a significant reduction in luciferase activity in the Si+LPS condition compared to the LPS-alone condition, indicating that Klf4 may be involved in regulating Cox-2 promoter activity. *, **, Statistical differences in comparison to control values and #, Statistical differences in comparison to LPS-treated values. (* p < 0.05, ** p < 0.01, # p < 0.01).

We have observed an increase in Cox-2 levels upon LPS treatment; therefore we investigated whether Klf4 regulates Cox-2 expression in microglial cells. RT-PCR for Cox-2 was carried out with RNA isolated from control, LPS, Si+LPS and Sc+LPS treated BV-2 cells. Interestingly, we observed a significant reduction of Cox-2 mRNA in Si+LPS cells when compared to LPS alone treated cells (p < 0.05) (Fig 6A). We also found a significant 2-fold reduction in Cox-2 protein levels in Si+LPS cells when compared to LPS treated samples (p < 0.01) (Fig 6B). Since the expression levels of Cox-2 decreased within 12 hours of LPS treatment upon Klf4 knockdown, it was likely that this transcription factor directly interacted with Cox-2 promoter in order to regulate and increase its expression levels. Interaction of Klf4 to Cox-2 promoter was evaluated using luciferase construct pCOX301-pGL2 which has Cox-2 promoter sequences upstream to firefly luciferase gene. We found a significant increase in luciferase activity in LPS and Sc+LPS cells (p < 0.01); however, in Si+LPS cells, luciferase activity significantly decreased by 8-fold with respect to LPS and Sc+LPS cells (p < 0.01) (Fig 6C). This finding suggests for the first time that Klf4 also interacts with Cox-2 promoter in addition to iNOS promoter in microglia upon endotoxin stimulation. However, since no reports indicate the known binding sequence of Klf4 on Cox-2 promoter, we could not confirm its interaction by performing EMSA using Cox-2 probes. It would be of interest to find out the possible binding site for Klf4 on Cox-2 promoters which would provide an insight into the mechanism of Klf4 in terms of Cox-2 regulation.

### Klf4 potentially interacts with pNF-kB

**Figure 7:**
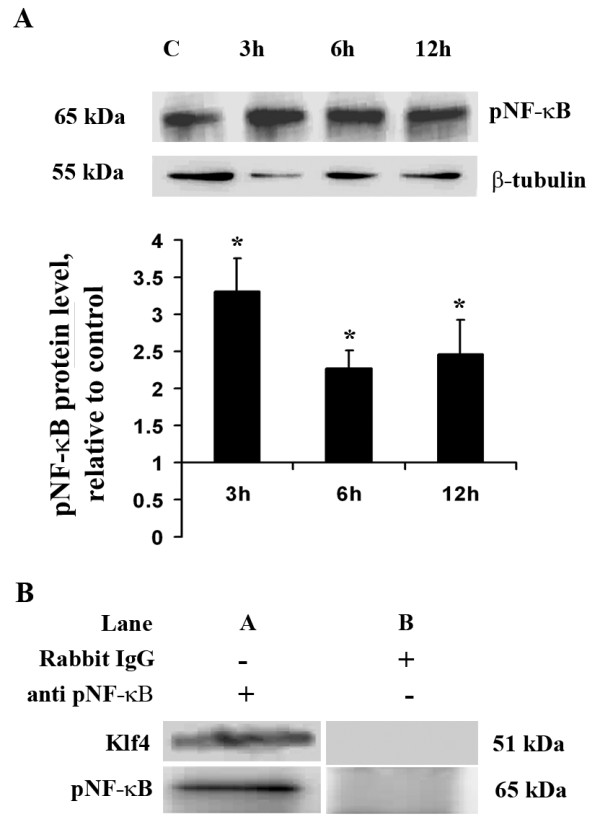
**Klf4 interacts with pNF-κB during inflammation**. **(A) **Immunoblot analysis of pNF-κB levels from total cellular extracts upon LPS treatment of BV-2 cells. The graph represents pNF-κB levels relative to untreated control sample at different time points. There is a significant increase in pNF-κB expression at all time points of LPS treatment compared to control. **(B) **Immunoblot and immunoprecipitation analysis of the interaction between pNF-κB and Klf4 in nuclear extracts of differently treated cells. Samples were 'pulled' down with a pNF-κB antibody. Lane A shows the blot for Klf4 in the upper panel and for pNF-κB in the lower panel in immunoprecipitate using anti-pNF-κB antibody, and lane B shows the same for immunoprecipitate using rabbit IgG as a control. *, **, Statistical differences in comparison to control values (* p < 0.05, ** p < 0.01).

Endotoxins such as LPS have been known to activate pNF-κB which mediates several pro-inflammatory pathways [11-13]. Klf4 binding sites have been reported to be closer to pNF-κB binding site on iNOS promoter and also that it interacts with pNF-κB [18]. Immunoblot from total cellular extracts from BV-2 cells treated with LPS for different time points of 3 h, 6 h and 12 h showed a significant increase in pNF-κB levels when compared to untreated control cells(p < 0.05) (Fig 7A). In order to investigate whether Klf4 interacts with pNF-κB in microglial cells upon LPS treatment, co-immunoprecipitation was carried out with 100 μg of nuclear extract using pNF-κB antibody and the blots were probed for Klf4 as well as for pNF-κB. We found that Klf4 was present in the immunoprecipitate that was prepared using pNF-κB antibody (Fig 7B). Rabbit IgG was used as a control for this experiment. From our findings we can infer that Klf4 interacts with pNF-κB upon LPS stimulation. Whether this interaction is crucial for transactivation of iNOS or Cox-2 promoters, a detailed study is further required.

## Discussion

Activation of microglia - which is marked by extensive proliferation, chemotaxis, and altered morphology - is the hallmark of neuroinflammation in several neurodegenerative diseases and pathological conditions of CNS. It's activation in response to LPS is well documented [44,45] and it generates numerous pro- and anti-inflammatory mediators upon activation including IL-1β, TNF-α, MCP-1 [9,46], IL-6 [47] and IL-10 [48] among other immunomodulators. From various studies on macrophages and microglia it is known that LPS binds to TLR4 receptors and initiates a cascade leading to the phosphorylation of NF-κB which plays a major role in inflammation in these cells [13,49]. However, inflammation is a complex process and, considering that several pathways are upregulated upon endotoxin stimulation, the role of other transcription factors and co-activators cannot be ruled out in LPS-induced inflammation. In this context, Klf4 has recently been identified as an inflammatory gene upregulated in macrophages during inflammation which has shown to be indispensable for iNOS expression in human macrophages [18]. However, reports on mouse macrophages have suggested that increased Klf4 expression in response to LPS is not associated with iNOS expression [30]. Since these studies were limited to peripheral system, it was important therefore to investigate the role of Klf4 in the activation of microglia to establish its involvement in neuroinflammation. Our studies have demonstrated for the first time in CNS that (i) Klf4 expression increases in microglial cells with time upon LPS stimulation *in vitro *and *in vivo*, (ii) Klf4 is involved in upregulation of pro-inflammatory cytokines and key inflammatory mediators including iNOS and Cox-2 and (iii) Klf4 interacts with pNF-κB upon LPS treatment for its pro-inflammatory activity.

At present, there are no known Klf4 knockout models available as Klf4^**-/- **^mice do not survive and die shortly after birth due to defect in epithelial differentiation and barrier formation [21,22]. Therefore, understanding the mechanism underlying the activation of Klf4 is limited to *in vitro *work. In tissues isolated from brains, we observed that Klf4 expression increases in response to LPS. Onset of inflammation in mouse brain was confirmed by an increase in pNF-κB and pro-inflammatory cytokines (data not shown) upon LPS treatment. We saw similar results in primary microglia isolated from P0-P2 BALB/c pups where LPS stimulation increased the Klf4 expression at earlier time points. A role for Klf4 in mediating inflammation could not be confirmed by these studies, which were limited to observing the expression of Klf4 in response to the bacterial endotoxin. We used BV-2 microglial cells to perform experiments where a transient knockdown was comparatively feasible to perform without any significant cell death. Our data suggest that expression of Klf4 increases upon LPS stimulation in a time and dose dependent manner in these microglial cells as well. Our observations for nuclear translocation studies suggest that Klf4 migrates to the nucleus upon endotoxin stimulation as early as 1 h after stimulation, and it is maintained at increased levels with respect to unstimulated control cells even at later time points. Keeping in mind the differences in either their cell cycle kinetics or response to inflammatory stimuli, minor discrepancies in the time kinetics of Klf4 nuclear localization between BV-2 cells and primary microglia were anticipated.

It is likely that Klf4 remains stored in the cytoplasm and its expression increases and it translocates to nucleus after LPS stimulation. Immunofluorescence images from BV-2 cells reveal that Klf4 is concentrated within specific regions of the nucleus which may be nucleoli [50]. So far, many Krüppel-type transcription factors have been found to be associated with nucleoli [51,52]. However, the roles of the nucleolar-targeted Krüppel-type zinc-finger proteins are not well known [50]. Considering the role of Klfs as transcription factors in acetylation reactions, it is also likely that they are localized into specialized regions of active transcription and are involved in differential acetylation of target genes [53]. Our findings indicate a possible role of Klf4 as a transcription factor in response to endotoxin stimulation. Most importantly, the expression of this transcription factor corresponded with that of key inflammatory enzymes, iNOS and Cox-2 as well as pro-inflammatory cytokines including TNF-α, MCP-1 and IL-6. Analysis of protein and tissues isolated from LPS treated mice also confirmed an increase in Klf4 with respect to control upon LPS administration.

iNOS is an important mediator of inflammation that catalyzes the production of nitric oxide (NO) which, under physiological conditions, can regulate vasoconstriction. NO also acts as a neurotransmitter in the CNS and has protective functions in anti-inflammatory pathways. However, at high concentrations NO readily reacts with superoxide ion (∙ O_2_^-^) to produce peroxynitrite (ONOO^-^) which causes irreversible damage to lipids and proteins resulting in cell death [54] and can therefore potentially be neurotoxic [55]. However, the mechanisms of neuronal death are complex and not very well understood [56]. The role of NF-κB in the LPS-mediated upregulation of iNOS is well established [5,16]. For the first time, we have shown that Klf4 is indispensable for iNOS expression in microglial cells. However, in agreement with studies on iNOS expression by Klf4 in human macrophages, we have shown a novel regulation of iNOS expression and subsequent NO production in microglia upon LPS stimulation. Klf4 knockdown resulted in decreased iNOS mRNA and protein expression within 12 hours of LPS treatment. This decrease in iNOS levels also correlated with a significant decrease in NO production observed in Si+LPS cells. Luciferase assays revealed that Klf4 interacts with iNOS promoter elements and upregulates iNOS expression upon LPS stimulation in microglial cells. Gel shift studies with biotinylated probe showed that Klf4 binds to -212 bp on iNOS promoter site in microglial cells upon LPS treatment. Given these observations, we suggest a possible role for Klf4 in iNOS expression and subsequent NO release in microglia and provide a novel mechanism for their upregulation in the presence of LPS. However, further studies are required in order to understand the detailed mechanism of Klf4 in iNOS-mediated neuronal death.

Cox-2 is another inflammatory enzyme, which catalyzes the rate-limiting step in the inducible production of prostaglandins like PGE2 [34] from arachidonic acid, which is then converted to active prostanoids by synthases [57]. The expression of Cox-2 requires binding of several cis-acting elements to Cox-2 promoters [58] and NF-κB plays a major role in the upregulation of Cox-2 and subsequent prostanoids generation [59-61]. The relationship between Cox-2 and Krüppel-like factors was not known until recent studies suggested that Cox-2 derived PGE2 may collaborate with Klf4 or Klf5 to regulate the activation of the complement system and exert diverse effects on the intestinal epithelium [62]. Since our findings suggest that Klf4 binds closer to the pNF-κB site on the iNOS promoter, and given that Cox-2 also has pNF-κB binding sequences on its promoter, we then studied the effect of Klf4 knockdown on Cox-2 expression. Interestingly, we saw a significant decrease in Cox-2 mRNA and protein levels within 12 h of LPS stimulation upon Klf4 knockdown. Promoter assays revealed that Klf4 interacts with Cox-2 promoters, which make Klf4 upregulation indispensable for Cox-2 expression in response to LPS stimulation. However, the exact sequences to which Klf4 binds on a Cox-2 promoter are not known. It would be of high interest to define possible binding sites for Klf4 on the Cox-2 promoter. Our finding is important considering the essential role played by Cox-2 in mediating neuroinflammation. However, we are not sure if Klf4 expression also mediates the production of PGE2 via Cox-2 upregulation. Its role in PGE2 production still needs to be determined.

All the existing studies have focused on the putative role of TNF-α, IFN-γ [18,30] and IL-1β [63] on the expression of Klf4. Moreover, the upregulation of several of these pro-inflammatory cytokines depends on NF-κB activation [13,49,64]. Therefore, in addition to understanding the role of Klf4 in mediating iNOS and COX-2 expression, we also determined its role in regulating the expression of pro-inflammatory cytokines. Our findings suggest that the expression of pro-inflammatory cytokines TNF-α, MCP-1 and IL-6 is also modulated by Klf4 in order to maintain a state of activation in microglial cells. We observed a significant decrease in the levels of these pro-inflammatory cytokines in Si+LPS cells which were significantly higher in LPS treated cells with respect to untreated controls. Further studies may be required in order to understand in detail the mechanism of Klf4-mediated cytokine release. These studies will provide a better understanding of how inflammation can be modulated by Klf4-mediated microglial activation. Overexpression studies using Klf4 constructs can also be carried out to further evaluate the extent of involvement of Klf4 in mediating inflammation.

Our findings prompt us to believe that Klf4 is a pleiotropic transcription factor which plays a key role in the establishment of neuroinflammation. The mechanisms of pro-inflammatory cytokine regulation by Klf4 are not clearly understood. Since pNF-κB is involved in the expression of these cytokines [5], it is likely that Klf4 acts as a binding partner to pNF-κB in co-operatively upregulating pro-inflammatory cytokines. Our co-immunoprecipitation studies confirm that Klf4 interacts with pNF-κB, and this result is in agreement with previously reported studies [18]. It is therefore possible that most of the pro-inflammatory activities that involve pNF-κB may have Klf4 as a potential binding partner and Klf4 activation might employ a similar pathway to that of pNF-κB. Given that Klf4 plays an important role in cell differentiation and proliferation, involvement of multiple pathways in Klf4 expression cannot be ruled out. Further studies, including overexpression of Klf4, will therefore be required for a full understanding of this transcription factor, which promises to be a potent target for therapeutic agents aiming to alleviate inflammation in brain.

## Competing interests

The authors declare that they have no competing interests.

## Authors' contributions

DKK designed and performed the experiments, analyzed the data and drafted the manuscript. SD and MG performed the experiments. AB participated in the design and coordination of the experiments. All the authors have reviewed the data and contributed to the preparation of the manuscript. All the authors have read and approved the final manuscript.

## References

[B1] Cerciat M Unkila M Garcia-Segura LM Arevalo MA Selective estrogen receptor modulators decrease the production of interleukin-6 and interferon-gamma-inducible protein-10 by astrocytes exposed to inflammatory challenge in vitro Glia 2010 58 93 102 10.1002/glia.20904 19533603

[B2] Carson MJ Thrash JC Lo D Analysis of microglial gene expression: identifying targets for CNS neurodegenerative and autoimmune disease Am J Pharmacogenomics 2004 4 321 30 10.2165/00129785-200404050-00005 15462610

[B3] Kreutzberg GW Microglia: a sensor for pathological events in the CNS Trends Neurosci 1996 19 312 8 10.1016/0166-2236(96)10049-7 8843599

[B4] Beyer M Gimsa U Eyupoglu IY Hailer NP Nitsch R Phagocytosis of neuronal or glial debris by microglial cells: upregulation of MHC class II expression and multinuclear giant cell formation in vitro Glia 2000 31 262 6 10.1002/1098-1136(200009)31:3<262::AID-GLIA70>3.0.CO;2-2 10941152

[B5] Brown GC Neher JJ Inflammatory Neurodegeneration and Mechanisms of Microglial Killing of Neurons Mol Neurobiol 2010 41 2-3 242 7 10.1007/s12035-010-8105-9 20195798

[B6] Perry VH Nicoll JA Holmes C Microglia in neurodegenerative disease Nat Rev Neurol 2010 6 193 201 10.1038/nrneurol.2010.17 20234358

[B7] Carson MJ Microglia as liaisons between the immune and central nervous systems: functional implications for multiple sclerosis Glia 2002 40 218 31 10.1002/glia.10145 12379909 PMC2693029

[B8] Kim YS Joh TH Microglia, major player in the brain inflammation: their roles in the pathogenesis of Parkinson's disease Exp Mol Med 2006 38 333 47 16953112 10.1038/emm.2006.40

[B9] Dinarello CA The interleukin-1 family: 10 years of discovery Faseb J 1994 8 1314 25 8001745

[B10] Takeuchi O Hoshino K Kawai T Sanjo H Takada H Ogawa T Takeda K Akira S Differential roles of TLR2 and TLR4 in recognition of gram-negative and gram-positive bacterial cell wall components Immunity 1999 11 443 51 10.1016/S1074-7613(00)80119-3 10549626

[B11] Qureshi ST Gros P Malo D Host resistance to infection: genetic control of lipopolysaccharide responsiveness by TOLL-like receptor genes Trends Genet 1999 15 291 4 10.1016/S0168-9525(99)01782-5 10431187

[B12] Zhang FX Kirschning CJ Mancinelli R Xu XP Jin Y Faure E Mantovani A Rothe M Muzio M Arditi M Bacterial lipopolysaccharide activates nuclear factor-kappaB through interleukin-1 signaling mediators in cultured human dermal endothelial cells and mononuclear phagocytes J Biol Chem 1999 274 7611 4 10.1074/jbc.274.12.7611 10075645

[B13] Doyle SL Jefferies CA O'Neill LA Bruton's tyrosine kinase is involved in p65-mediated transactivation and phosphorylation of p65 on serine 536 during NFkappaB activation by lipopolysaccharide J Biol Chem 2005 280 23496 501 10.1074/jbc.C500053200 15849198

[B14] Surh YJ Chun KS Cha HH Han SS Keum YS Park KK Lee SS Molecular mechanisms underlying chemopreventive activities of anti-inflammatory phytochemicals: down-regulation of COX-2 and iNOS through suppression of NF-kappa B activation Mutat Res 2001 480-481 243 68 11506818 10.1016/s0027-5107(01)00183-x

[B15] Rhee SH Hwang D Murine TOLL-like receptor 4 confers lipopolysaccharide responsiveness as determined by activation of NF kappa B and expression of the inducible cyclooxygenase J Biol Chem 2000 275 34035 40 10.1074/jbc.M007386200 10952994

[B16] Shen S Yu S Binek J Chalimoniuk M Zhang X Lo SC Hannink M Wu J Fritsche K Donato R Sun GY Distinct signaling pathways for induction of type II NOS by IFNgamma and LPS in BV-2 microglial cells Neurochem Int 2005 47 298 307 10.1016/j.neuint.2005.03.007 15955597

[B17] SenBanerjee S Lin Z Atkins GB Greif DM Rao RM Kumar A Feinberg MW Chen Z Simon DI Luscinskas FW Michel TM Gimbrone MA Jr Garcia-Cardena G Jain MK KLF2 Is a novel transcriptional regulator of endothelial proinflammatory activation J Exp Med 2004 199 1305 15 10.1084/jem.20031132 15136591 PMC2211816

[B18] Feinberg MW Cao Z Wara AK Lebedeva MA Senbanerjee S Jain MK Kruppel-like factor 4 is a mediator of proinflammatory signaling in macrophages J Biol Chem 2005 280 38247 58 10.1074/jbc.M509378200 16169848

[B19] Hamik A Lin Z Kumar A Balcells M Sinha S Katz J Feinberg MW Gerzsten RE Edelman ER Jain MK Kruppel-like factor 4 regulates endothelial inflammation J Biol Chem 2007 282 13769 79 10.1074/jbc.M700078200 17339326

[B20] Shields JM Christy RJ Yang VW Identification and characterization of a gene encoding a gut-enriched Kruppel-like factor expressed during growth arrest J Biol Chem 1996 271 20009 17 10.1074/jbc.271.33.20009 8702718 PMC2330254

[B21] Katz JP Perreault N Goldstein BG Lee CS Labosky PA Yang VW Kaestner KH The zinc-finger transcription factor Klf4 is required for terminal differentiation of goblet cells in the colon Development 2002 129 2619 28 12015290 10.1242/dev.129.11.2619 PMC2225535

[B22] Segre JA Bauer C Fuchs E Klf4 is a transcription factor required for establishing the barrier function of the skin Nat Genet 1999 22 356 60 10.1038/11926 10431239

[B23] Yoon HS Chen X Yang VW Kruppel-like factor 4 mediates p53-dependent G1/S cell cycle arrest in response to DNA damage J Biol Chem 2003 278 2101 5 10.1074/jbc.M211027200 12427745 PMC2229830

[B24] Yoon HS Yang VW Requirement of Kruppel-like factor 4 in preventing entry into mitosis following DNA damage J Biol Chem 2004 279 5035 41 10.1074/jbc.M307631200 14627709 PMC1262649

[B25] Li Y McClintick J Zhong L Edenberg HJ Yoder MC Chan RJ Murine embryonic stem cell differentiation is promoted by SOCS-3 and inhibited by the zinc finger transcription factor Klf4 Blood 2005 105 635 7 10.1182/blood-2004-07-2681 15358627

[B26] Takahashi K Yamanaka S Induction of pluripotent stem cells from mouse embryonic and adult fibroblast cultures by defined factors Cell 2006 126 663 76 10.1016/j.cell.2006.07.024 16904174

[B27] Kuhn DM Francescutti-Verbeem DM Thomas DM Dopamine quinones activate microglia and induce a neurotoxic gene expression profile: relationship to methamphetamine-induced nerve ending damage Ann N Y Acad Sci 2006 1074 31 41 10.1196/annals.1369.003 17105901

[B28] Liu J Liu Y Zhang H Chen G Wang K Xiao X KLF4 promotes the expression, translocation, and releas eof HMGB1 in RAW264.7 macrophages in response to LPS Shock 2008 30 260 6 18197146 10.1097/shk.0b013e318162bef7

[B29] Shen B Smith RS Jr Hsu YT Chao L Chao J Kruppel-like factor 4 is a novel mediator of Kallistatin in inhibiting endothelial inflammation via increased endothelial nitric-oxide synthase expression J Biol Chem 2009 284 35471 8 10.1074/jbc.M109.046813 19858207 PMC2790976

[B30] Alder JK Georgantas RW Hildreth RL Kaplan IM Morisot S Yu X McDevitt M Civin CI Kruppel-like factor 4 is essential for inflammatory monocyte differentiation in vivo J Immunol 2008 180 5645 52 18390749 10.4049/jimmunol.180.8.5645 PMC3074963

[B31] Dutta K Ghosh D Nazmi A Kumawat KL Basu A A common carcinogen benzo[a]pyrene causes neuronal death in mouse via microglial activation PLoS One 2010 5 e9984 10.1371/journal.pone.0009984 20376308 PMC2848611

[B32] Basu A Krady JK Enterline JR Levison SW Transforming growth factor beta1 prevents IL-1beta-induced microglial activation, whereas TNFalpha- and IL-6-stimulated activation are not antagonized Glia 2002 40 109 20 10.1002/glia.10118 12237848

[B33] Perrella MA Patterson C Tan L Yet SF Hsieh CM Yoshizumi M Lee ME Suppression of interleukin-1beta-induced nitric-oxide synthase promoter/enhancer activity by transforming growth factor-beta1 in vascular smooth muscle cells. Evidence for mechanisms other than NF-kappaB J Biol Chem 1996 271 13776 80 10.1074/jbc.271.23.13776 8662809

[B34] Pathak SK Bhattacharyya A Pathak S Basak C Mandal D Kundu M Basu J Toll-like receptor 2 and mitogen- and stress-activated kinase 1 are effectors of Mycobacterium avium-induced cyclooxygenase-2 expression in macrophages J Biol Chem 2004 279 55127 36 10.1074/jbc.M409885200 15496409

[B35] Ghoshal A Das S Ghosh S Mishra MK Sharma V Koli P Sen E Basu A Proinflammatory mediators released by activated microglia induces neuronal death in Japanese encephalitis Glia 2007 55 483 96 10.1002/glia.20474 17203475

[B36] Das S Ghosh D Basu A Japanese encephalitis virus induce immuno-competency in neural stem/progenitor cells PLoS One 2009 4 e8134 10.1371/journal.pone.0008134 19956550 PMC2780913

[B37] Livak KJ Schmittgen TD Analysis of relative gene expression data using real-time quantitative PCR and the 2(-Delta Delta C(T)) Method Methods 2001 25 402 8 10.1006/meth.2001.1262 11846609

[B38] Soldan SS Alvarez Retuerto AI Sicotte NL Voskuhl RR Dysregulation of IL-10 and IL-12p40 in secondary progressive multiple sclerosis J Neuroimmunol 2004 146 209 15 10.1016/j.jneuroim.2003.10.033 14698865

[B39] Shukla S Gupta S Suppression of constitutive and tumor necrosis factor alpha-induced nuclear factor (NF)-kappaB activation and induction of apoptosis by apigenin in human prostate carcinoma PC-3 cells: correlation with down-regulation of NF-kappaB-responsive genes Clin Cancer Res 2004 10 3169 78 10.1158/1078-0432.CCR-03-0586 15131058

[B40] Eugenin EA Berman JW Chemokine-dependent mechanisms of leukocyte trafficking across a model of the blood-brain barrier Methods 2003 29 351 61 10.1016/S1046-2023(02)00359-6 12725802

[B41] Akira S Taga T Kishimoto T Interleukin-6 in biology and medicine Adv Immunol 1993 54 1 78 full_text 8379461 10.1016/s0065-2776(08)60532-5

[B42] Kishimoto T Interleukin-6 and its receptor in autoimmunity J Autoimmun 1992 5 Suppl A 123 32 10.1016/0896-8411(92)90027-N 1380241

[B43] Fialkow L Wang Y Downey GP Reactive oxygen and nitrogen species as signaling molecules regulating neutrophil function Free Radic Biol Med 2007 42 153 64 10.1016/j.freeradbiomed.2006.09.030 17189821

[B44] Minghetti L Ajmone-Cat MA De Berardinis MA De Simone R Microglial activation in chronic neurodegenerative diseases: roles of apoptotic neurons and chronic stimulation Brain Res Brain Res Rev 2005 48 251 6 10.1016/j.brainresrev.2004.12.015 15850664

[B45] Waetzig V Czeloth K Hidding U Mielke K Kanzow M Brecht S Goetz M Lucius R Herdegen T Hanisch UK c-Jun N-terminal kinases (JNKs) mediate pro-inflammatory actions of microglia Glia 2005 50 235 46 10.1002/glia.20173 15739188

[B46] Qin L Wu X Block ML Liu Y Breese GR Hong JS Knapp DJ Crews FT Systemic LPS causes chronic neuroinflammation and progressive neurodegeneration Glia 2007 55 453 62 10.1002/glia.20467 17203472 PMC2871685

[B47] Lund S Christensen KV Hedtjarn M Mortensen AL Hagberg H Falsig J Hasseldam H Schrattenholz A Porzgen P Leist M The dynamics of the LPS triggered inflammatory response of murine microglia under different culture and in vivo conditions J Neuroimmunol 2006 180 71 87 10.1016/j.jneuroim.2006.07.007 16996144

[B48] Ledeboer A Breve JJ Wierinckx A van der Jagt S Bristow AF Leysen JE Tilders FJ Van Dam AM Expression and regulation of interleukin-10 and interleukin-10 receptor in rat astroglial and microglial cells Eur J Neurosci 2002 16 1175 85 10.1046/j.1460-9568.2002.02200.x 12405978

[B49] Sakurai H Chiba H Miyoshi H Sugita T Toriumi W IkappaB kinases phosphorylate NF-kappaB p65 subunit on serine 536 in the transactivation domain J Biol Chem 1999 274 30353 6 10.1074/jbc.274.43.30353 10521409

[B50] Medugno L Florio F Cesaro E Grosso M Lupo A Izzo P Costanzo P Differential expression and cellular localization of ZNF224 and ZNF255, two isoforms of the Kruppel-like zinc-finger protein family Gene 2007 403 125 31 10.1016/j.gene.2007.07.036 17900823

[B51] Huang Z Philippin B O'Leary E Bonventre JV Kriz W Witzgall R Expression of the transcriptional repressor protein Kid-1 leads to the disintegration of the nucleolus J Biol Chem 1999 274 7640 8 10.1074/jbc.274.12.7640 10075651

[B52] Yano K Ueki N Oda T Seki N Masuho Y Muramatsu M Identification and characterization of human ZNF274 cDNA, which encodes a novel kruppel-type zinc-finger protein having nucleolar targeting ability Genomics 2000 65 75 80 10.1006/geno.2000.6140 10777669

[B53] Evans PM Zhang W Chen X Yang J Bhakat KK Liu C Kruppel-like factor 4 is acetylated by p300 and regulates gene transcription via modulation of histone acetylation J Biol Chem 2007 282 33994 4002 10.1074/jbc.M701847200 17908689

[B54] Iadecola C Zhang F Permissive and obligatory roles of NO in cerebrovascular responses to hypercapnia and acetylcholine Am J Physiol 1996 271 R990 1001 8897992 10.1152/ajpregu.1996.271.4.R990

[B55] Brown GC Cooper CE Nanomolar concentrations of nitric oxide reversibly inhibit synaptosomal respiration by competing with oxygen at cytochrome oxidase FEBS Lett 1994 356 295 8 10.1016/0014-5793(94)01290-3 7805858

[B56] Block ML Zecca L Hong JS Microglia-mediated neurotoxicity: uncovering the molecular mechanisms Nat Rev Neurosci 2007 8 57 69 10.1038/nrn2038 17180163

[B57] Herschman HR Prostaglandin synthase 2 Biochim Biophys Acta 1996 1299 125 40 8555245 10.1016/0005-2760(95)00194-8

[B58] Tanabe T Tohnai N Cyclooxygenase isozymes and their gene structures and expression Prostaglandins Other Lipid Mediat 2002 68-69 95 114 10.1016/S0090-6980(02)00024-2 12432912

[B59] Mestre JR Mackrell PJ Rivadeneira DE Stapleton PP Tanabe T Daly JM Redundancy in the signaling pathways and promoter elements regulating cyclooxygenase-2 gene expression in endotoxin-treated macrophage/monocytic cells J Biol Chem 2001 276 3977 82 10.1074/jbc.M005077200 11092878

[B60] de Oliveira AC Candelario-Jalil E Bhatia HS Lieb K Hull M Fiebich BL Regulation of prostaglandin E2 synthase expression in activated primary rat microglia: evidence for uncoupled regulation of mPGES-1 and COX-2 Glia 2008 56 844 55 10.1002/glia.20658 18383341

[B61] Caivano M Cohen P Role of mitogen-activated protein kinase cascades in mediating lipopolysaccharide-stimulated induction of cyclooxygenase-2 and IL-1 beta in RAW264 macrophages J Immunol 2000 164 3018 25 10706690 10.4049/jimmunol.164.6.3018

[B62] Shao J Yang VW Sheng H Prostaglandin E2 and Kruppel-like transcription factors synergistically induce the expression of decay-accelerating factor in intestinal epithelial cells Immunology 2008 125 397 407 10.1111/j.1365-2567.2008.02847.x 18435741 PMC2669143

[B63] Cullingford TE Butler MJ Marshall AK Tham el L Sugden PH Clerk A Differential regulation of Kruppel-like factor family transcription factor expression in neonatal rat cardiac myocytes: effects of endothelin-1, oxidative stress and cytokines Biochim Biophys Acta 2008 1783 1229 36 10.1016/j.bbamcr.2008.03.007 18406357 PMC2396231

[B64] Petrova TV Akama KT Van Eldik LJ Cyclopentenone prostaglandins suppress activation of microglia: down-regulation of inducible nitric-oxide synthase by 15-deoxy-Delta12,14-prostaglandin J2 Proc Natl Acad Sci USA 1999 96 4668 73 10.1073/pnas.96.8.4668 10200320 PMC16390

